# Preserving professional identities, behaviors, and values in digital professionalism using social networking sites; a systematic review

**DOI:** 10.1186/s12909-021-02802-9

**Published:** 2021-07-12

**Authors:** Shaista Salman Guraya, Salman Yousuf Guraya, Muhamad Saiful Bahri Yusoff

**Affiliations:** 1 Royal College of Surgeons Ireland, RCSI - MUB, Busaiteen, Bahrain; 2 Department of Medical Education, School of Medical Sciences, University Sains Malaysia, Health campus, Kelantan Kota Bahru, Malaysia; 3 Clinical Sciences Department, College of Medicine, University of Sharjah, Sharjah, United Arab Emirates

**Keywords:** Professionalism, Digital professionalism, Professional identity, Professional behaviors, Professional values, Professional ethics, Social media, Social networking sites, Health professionals

## Abstract

**Background:**

Despite a rapid rise of use of social media in medical disciplines, uncertainty prevails among healthcare professionals for providing medical content on social media. There are also growing concerns about unprofessional behaviors and blurring of professional identities that are undermining digital professionalism. This review tapped the literature to determine the impact of social media on medical professionalism and how can professional identities and values be maintained in digital era.

**Methods:**

We searched the databases of PubMed, ProQuest, ScienceDirect, Web of Science, and EBSCO host using (professionalism AND (professionalism OR (professional identity) OR (professional behaviors) OR (professional values) OR (professional ethics))) AND ((social media) AND ((social media) OR (social networking sites) OR Twitter OR Facebook)) AND (health professionals). The research questions were based on sample (health professionals), phenomenon of interest (digital professionalism), design, evaluation and research type. We screened initial yield of titles using pre-determined inclusion and exclusion criteria and selected a group of articles for qualitative analysis. We used the Biblioshiny® software package for the generation of popular concepts as clustered keywords.

**Results:**

Our search yielded 44 articles with four leading themes; marked rise in the use of social media by healthcare professionals and students, negative impact of social media on digital professionalism, blurring of medical professional values, behaviors, and identity in the digital era, and limited evidence for teaching and assessing digital professionalism. A high occurrence of violation of patient privacy, professional integrity and cyberbullying were identified. Our search revealed a paucity of existing guidelines and policies for digital professionalism that can safeguard healthcare professionals, students and patients.

**Conclusions:**

Our systematic review reports a significant rise of unprofessional behaviors in social media among healthcare professionals. We could not identify the desired professional behaviors and values essential for digital identity formation. The boundaries between personal and professional practices are mystified in digital professionalism. These findings call for potential educational ramifications to resurrect professional virtues, behaviors and identities of healthcare professionals and students.

**Supplementary Information:**

The online version contains supplementary material available at 10.1186/s12909-021-02802-9.

## Background

Social media is based on a collection of digital platforms whose content is created, edited and shared by its clients themselves [1]. The expeditious development of social media has transformed the way healthcare professionals and students interact with each other [2]. Facebook, Twitter, LinkedIn, YouTube, Instagram, Wikis, Blogs, Podcasts and WeChat are the most popular social media worldwide [3]. Medical professionalism is a multi-dimensional construct that refers to a set of skills and competencies that the professionals are expected to practice [4] The crossroads of medical professionalism and the use of social media has created a new facet of digital professionalism, interchangeable with e-professionalism, that reflects the manifestation of traditional professional attitudes and behaviors through social media [5]. Digital professionalism refers to the professionals’ use of digital media and the mechanisms in which the profession is evolved by this use [6].

The concept of digital professionalism in e-health embraces the core values that can steer teaching, learning and practice domains in medical disciplines through online platforms. A safe application of digital professionalism includes professional competence, reputation, and responsibility [7]. Digital media provides enormous interconnectivity that has expanded our range of opportunities for sharing information. However, this unprecedented opportunity has created interdependency on social media, devices, and users with loss of natural pauses for self-reflection in our livelihood. The ubiquity and easy access of digital media permits free communications that has the potential to thrust its contents into the medical practice.

The fluid and complex nature of medical professional virtues, behaviours and identities are more vulnerable in the current era of digital professionalism [8]. Professional virtues and behaviours illustrate the processes of how the professionals enact their role, while professional identity involves an oath for adhering to the values and ethics of medical profession associated with the profession such as being trustworthy, competent, and safe medical practitioner. Medical professional identity requires the practicing physician to act as a professional at individual, interpersonal and societal levels [9]. On the hand, digital professional identity pertains to a wide range of distinct personal and professional acts that are manifested in the digital space [10]. Unfortunately, literature has reported erosions of professional identities and behaviours in the current era of digital professionalism [11]. Inappropriate social media behavior has also shown detrimental effects on medical and health sciences students’ approach towards humanism, empathy and altruism [5]. Unauthorized postings of patient health information, pictures, patient-doctor communication blogs, and images with clear patient identification are commonly witnessed unprofessional behaviors. This practice has blurred personal and professional boundaries in the medical sphere. In digital professionalism, medical educators and policymakers are skeptical about preserving patient confidentiality and privacy on social media [12].

There are growing concerns about the absence of a structured program for digital professionalism in the medical and health sciences [13, 14]. In addition, there is a paucity of literature that can help understand the mechanisms for safe-guarding medical professionals’ identities and values in the digital world [15]. This systematic review aimed to review the available body of knowledge that can help identify key concepts and threats to professional identity in the era of digital professionalism.

## Methods

### Research questions

Our research questions were based on Sample, Phenomenon of Interest, Design, Evaluation and Research type (SPIDER) [16] as shown in Table 1.

**Table 1:** Selection criteria for the studies in this systematic review using SPIDER (*n* = 44)

Variables	Inclusion criteria
**Sample**	**-** Health professionals -Medical and nursing undergraduate and postgraduate students and/or residents -Physicians and fellows -Medical educators or school administrators -Medical school websites -Respondents from medicine, pharmacy, dentistry, and nursing
**Phenomenon of Interest**	Digital professionalism, Professional behaviors, attitudes and identity in digital world
**Design**	Questionnaire/Survey Interview Focus group Observational studies Ethnography Content analysis
**Evaluation**	Views Experiences Opinions/Attitudes/Perceptions/Beliefs/Ideas Knowledge/Understanding Behaviours
**Research type**	-Qualitative -Quantitative -Mixed method

*S* Sample; *PI* Phenomenon of Interest; *D* Design; *E* Evaluation; *R* Research type

We framed the following specific questions for our systematic review;

I. What are the desired values and behaviors of digital professionalism that are needed for maintaining digital professional identity?
II. What is the impact of social media on medical professionalism?
III. How can values and behaviors of digital professionalism be made measurable and reproducible in teaching and assessment?

### Search strategy

In May 2020, we searched the databases of PubMed, ProQuest, ScienceDirect, Web of Science, and EBSCO host using keywords (professionalism AND (professionalism OR (professional identity) OR (professional behaviors) OR (professional values) OR (professional ethics))) AND ((social media) AND ((social media) OR (social networking sites) OR Twitter OR Facebook)) AND (health professionals) terms and text words for the English language articles published during 1st January 2015 till 30th April, 2020. The search focused on titles about definitions, analyses and relationships of health professionals about professional identity, virtues, behaviors, medical professionalism and digital professionalism.

PubMed was the mainstay to systematically develop a search string, which was later extrapolated to other databases. All selected keywords were searched in the fields “Abstract” and “Article Title” (alternatively “Topic”) and in MeSH/Subject Headings/Thesaurus where available. Language, document type, and publication year restrictions were instead included in the exclusion criteria for the screening process. We defined healthcare professionals in undergraduate, graduate, and continuing education, postgraduates and practicing physicians/nurses, deans, directors, and faculty. For this study we defined healthcare professionals as individuals who may be involved in healthcare delivery (for example: physicians, nurses, dentists, physiotherapists, and pharmacists). A full search log, including detailed search strings for all included information sources, results and notes is available in Appendix I.

### Data collection, eligibility criteria and the selection of articles

We used the Preferred Reporting Items for Systematic Reviews and Meta-analyses (PRISMA) guidelines was used for data mining and selection of the studies for this systematic review [17].

The original research articles that conducted qualitative, quantitative and mixed methods studies about definitions of digital professionalism, e-professionalism in the digital age, guidelines for the usage of social media, and the degree and extent of usage of social media by health professionals for educational, professional and personal purposes were included. The participants of the selected studies were medical and allied health sciences students, physicians, faculty and program directors. We excluded systematic reviews, meta-analysis, editorials, and commentaries from our search. The studies about professionalism in non-medical fields were also excluded.

SS reviewed the titles and abstracts of the studies retrieved during initial search and grouped relevant articles for possible inclusion. Then we reviewed full text of the selected articles for their further matching with the inclusion criteria. To mitigate research bias, the entire search process was reviewed by SS, SYG and MSBY. We resolved research disagreements and disputes through discussions until we reached a consensus.

### Data extraction and synthesis

This step included review of information from the articles, publication year, author, country of study, single center/multicenter, study level, health professionals’ discipline, ethical approval, methodology, study purpose, results and Medical Education Research Study Quality Instrument (MERSQI) [18] score (Appendix II)**.** The data was organized in charts for the descriptive analysis of the quantity and quality of the selected studies.

We performed thematic analysis using emerging concepts and theories from the selected studies, which generated different concepts. The leading themes and concepts were further analyzed in discussion to reach consensus for future implementations. We coded the findings of the selected articles and constructed a coding tree. Later, all researchers critically analyzed preliminary themes, which refined the coding process and helped in adding more strings such as assessment and policy about digital professionalism. We also used biblioshiny® from R Statistical Package to carry out bibliometric analysis [19]. Using the hierarchical clustering strategy, we labelled each keyword as a cluster item, and then merged clusters with maximum similarity into a large new cluster. Finally, the multiple cluster analysis was graphically generated for review.

### Quality assessment

We used the MERSQI tool for the evaluation of studies of quantitative educational research. The MERSQI checklist has 10 items in six domains: study design, sampling, type of data, validity evidence, data analysis, and type of outcomes with a maximum score of three in each domain. A study can have a maximum MERSQI score of 18 (highest quality). SS individually scored each study and in case of score discrepancies, SYG re-assessed the scoring and the results were cross verified among researchers.

### Quality assurance

All researchers (SS, SYG and MSBY) objectively reviewed the workflow for the selection of studies. In case of discrepancies, the researchers reached consensus by comparing the studies with inclusion criteria and key words. The discrepancies, inconsistencies and controversies were resolved with consensus until all the concerns were resolved.

## Results

**Fig. 1:**
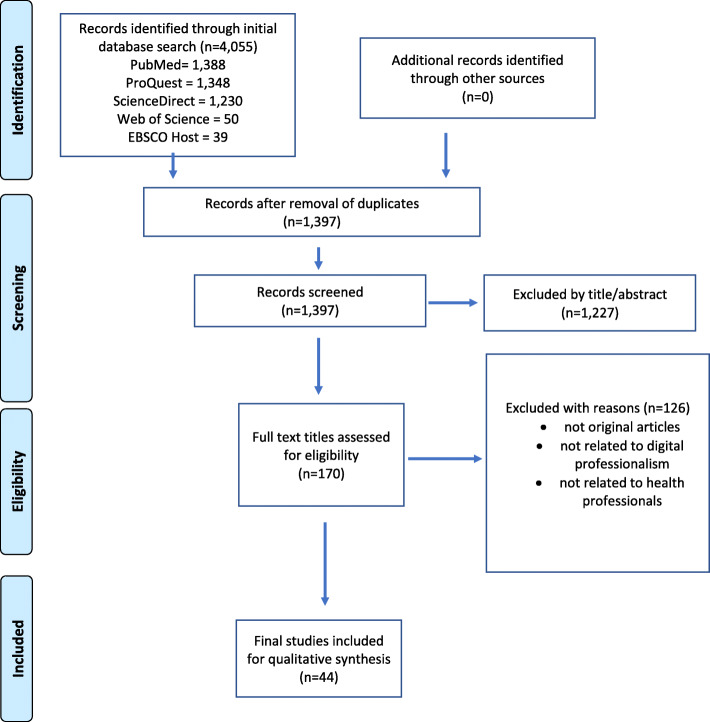
The Preferred Reporting Items for Systematic Reviews and Meta-Analysis (PRISMA) flow diagram for the selection of studies in this systematic review

Initial search retrieved 4,055 titles, and after eliminating duplicates and retaining only English language publications, we included 1,319 for further abstract analysis. During the exclusion phase, 1,277 titles were excluded as they could not meet the inclusion criteria. Lastly, 126 full text articles were excluded from the remaining 170 publications. After full paper review, we included 44 articles in our systematic review for deeper analysis. The entire process using PRISMA guidelines is illustrated in Fig. 1.

**Fig. 2:**
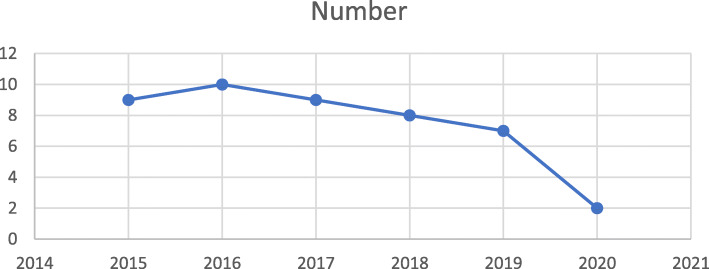
The yearly publication pattern of articles about professional identity, behaviors and virtues in the digital world during 2015–2020 (*n* = 44). This search was conducted in May 2020, which explains lower number of articles in 2020

The yearly publication pattern of the selected 44 articles about professional identity, behaviors and virtues in the digital world is shown in Fig. 2. A maximum number of 10 articles were published in 2016.

**Fig. 3:**
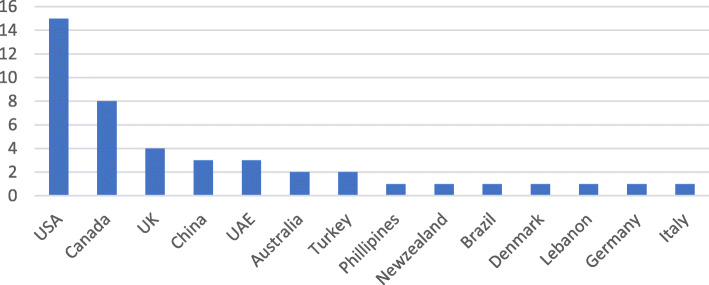
The country-wise pattern of articles published about professional identity, behaviors and virtues in the digital world during 2015–2020 (*n* = 44)

From a different perspective, the graphical representation of countries of origin of the selected 44 studies is displayed in Fig. 3.

Most studies were based in the USA (15/34 %), while other studies were based in Canada (8/18 %), UK (4/9 %), China (3/7 %), UAE (3/7 %), and New Zealand (1/3 %). Most commonly used methodologies were cross-sectional surveys (27/61 %) and analysis of the publicly available Internet content such as Facebook profiles, Twitter streams, or blogs (8/18 %). Other methods used in the selected studies included focus group discussions, mixed-methods by semi-structured interviews and survey. Of note, of all the survey-based studies, about half of these studies had response rates of 50 % or greater, while three studies either did not explicitly report a response rate. For the 11 studies that analyzed the publicly available Internet content, four (36 %) did not mention any methods to increase study rigor of data extraction and analysis expected of content analyses.

Interestingly, 32 (73 %) studies had clear ethical statements either with institutional board approval, exemption, or undertaking that ethical approval was not necessary. Table 2 shows descriptive analysis of the data about medical disciplines and study levels from the selected 44 studies in this systematic review. In terms of study populations, 23 (52 %) involved postgraduates and/or residents, physicians and fellows, and practising nurses, 18 (41 %) included undergraduate students (medical, dental or nursing) while 7 (6 %) conducted studies involved dean, directors, and faculty. Approximately 50 % of survey-based studies had response rates of 50 % or more, and postgraduates & practicing physicians/nurses were the most common group of studied participants.

**Table 2:** Descriptive analysis of the data about medical disciplines and study levels from the selected studies in this systematic review (n-44)

Features	Analysis	No.
**Disciplines**	Medicine Nursing Dentistry Pharmacy Physiotherapy	27 9 5 2 1
**Study level**	Postgraduates & Practicing physicians/nurses Undergraduate Dean, directors, and faculty	23 18 7

**Fig. 4:**
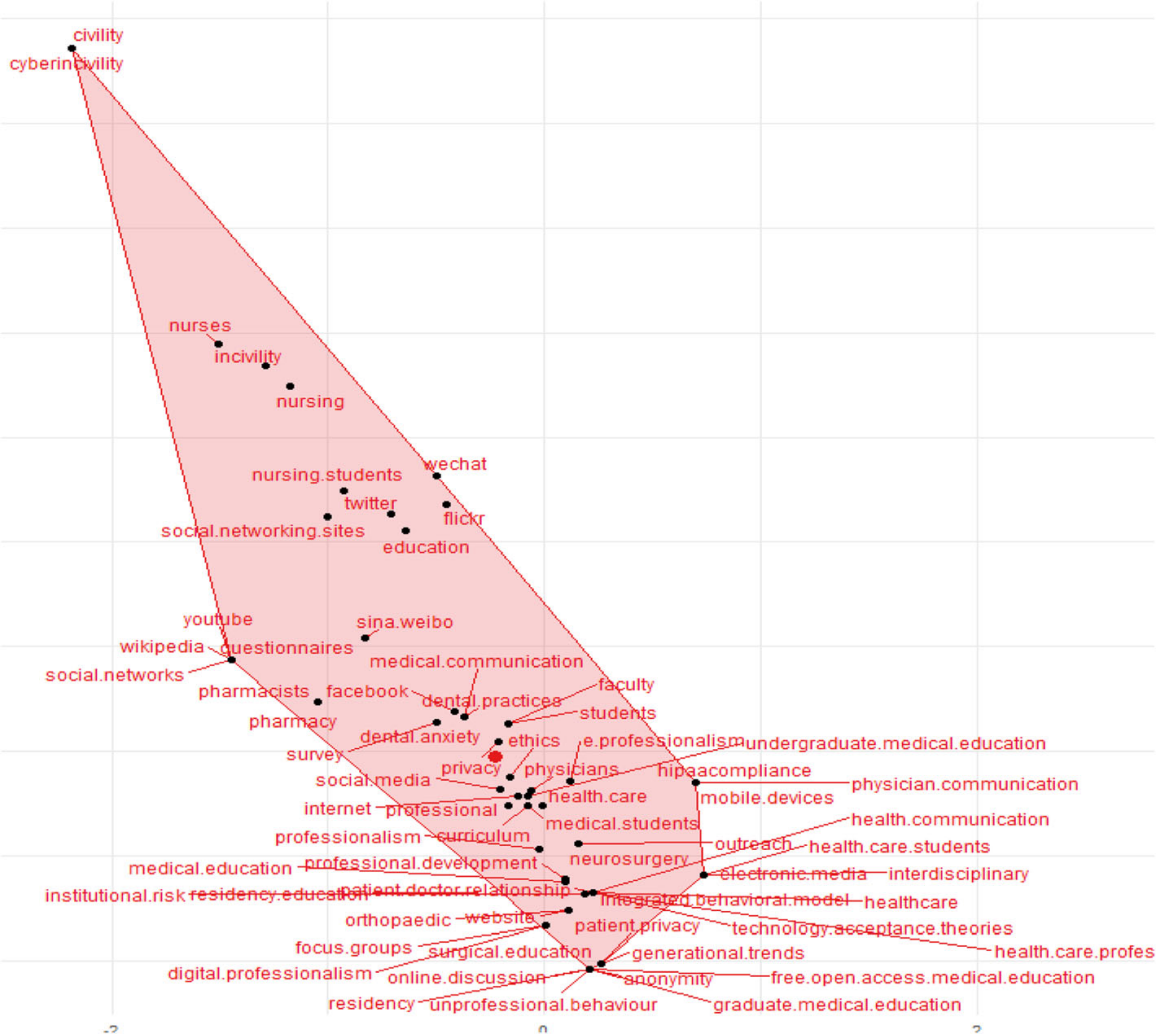
Bibliometric analysis illustrating the cluster and multiple interconnections of frequently used keywords

A graphical relationship among the selected keywords for our systematic review using the bibliometric analysis is illustrated in Fig. 4. The plane distance between keywords reflects the degree of similarity and commonalities among them. The keywords approaching centre of the figure indicate that they have received high attention in the recent years. Probity received maximum attention, while cybercivilty received least attention and similarity with other keywords.

The analysis of MERSQI showed that 32 quantitative studies had average score of 12, while other 12 qualitative studies did not qualify for quality check. A maximum number of 14 studies had a primary research objective of exploring the beliefs and attitudes of the participants towards usage of social media use and professional behaviours. Other leading research objectives of the selected studies in our systematic review are outlined in Table 3.

**Table 3:** Leading research purposes of the selected studies in this systematic review (*n*-44)

Study purposes	*n* = 44
Explore beliefs and attitudes regarding social media use and professional behaviours	14
Quantify and evaluate professional digital media use	7
Describe professional and personal information and activities, perceptions of online professional behavior and opinions on guidelines in this area.	5
Examine the effects of an educational intervention to assess students’ change in social media use practices	4
Determine educational use of Social media	3
Identify and characterize the types of unprofessional and concomitant personal and institutional risks.	3
Describe appropriate patient-physician relationship on social media.	3
Describe the characteristics of professional/unprofessional online posts or tweets	2
Describe perceptions of confidentiality, accountability, and e-professionalism	2
Describe relationship between anonymity and professionalism	1

### Our systematic review generated four main themes;

I. Usage of social media by healthcare professionals and students [1, 3, 11, 20–42].
II. The impact of social media on medical professionalism [20, 25–29, 31, 33–35, 37, 40, 41, 43–50].
III. Blurring of professional values, behaviors, and identity in the digital era [5, 11, 20, 22, 26, 28, 29, 31, 32, 35, 37, 42, 43, 46, 48, 50–56].
IV. Limited evidence for teaching and assessing professionalism in the digital era [5, 11, 20, 27, 29, 31, 32, 34, 42, 48, 49, 54, 55].

By and large, the usage of social media by health professionals has escalated during the last decade [1, 3, 11, 20–42, 45–49], there is a negative impact of social media usage on medical professionalism as reflected by erosion of professional integrity [20, 25, 29, 33–35], an upsurge of awareness about professional identity but rise in unprofessional behaviors in the digital era [11, 20, 22, 29, 32, 35, 47, 48, 51, 52, 57] and some evidence of enhanced acquisition of knowledge about digital professionalism by incorporating structured modules in curricula [5, 20, 27, 29, 32, 34, 42, 48, 49, 54, 55].

## Discussion

This systematic review reports a rapid rise in the usage of social media by healthcare professionals and students with a negative impact of social media as reflected by substantial unprofessional behaviors leading to blurred professional identities. There is a compelling evidence that the awareness of social media by healthcare professionals and students is getting better, nevertheless, there is a reciprocal increase in the prevalence of unprofessional behaviors in the digital era. We could find limited and unsatisfactory data about the appropriate acquisition of knowledge and structured curriculum for teaching and assessment of digital professionalism. Unfortunately, this review could not identify the desired values and behaviors of digital professionalism that are needed for maintaining digital professional identity. Of all traits of medical professionalism, probity found highest attention in the studies selected in our systematic review. Probity in medical disciplines is an ever needed professional characteristic that enriches the faculty-student and physician-patient relationships with elements of honesty and trust [58].

The four leading themes of this systematic review are elaborated in the following parts of discussion.

### Theme I: Usage of social media by health professionals and students

The use of social media is among the most innovative but, unfortunately, the most destructive necessary evil of the current era. Currently, more than 40 % of the health care consumers use social media for their healthcare needs worldwide [59]. In medical education, social media is being increasingly used for learning and teaching, research, hospital care quality, and for assessment of online behaviour of healthcare professionals [60]. Only in the USA, nearly 65 % of the adult population use social media for different reasons and this usage has sharply risen in the last decade [61]. Understandably this usage is ubiquitous among young adults (90 %) and notable among older adults (77 %), this difference being reflected by being digital native and digital immigrants, respectively.

In medical education, 94 % of medical students, 79 % of medical residents, and 42 % of practicing physicians use social media [62]. This exponential growth in social media usage provides health information, facilitate live chat platforms for patient-to-patient and patient-to-health professional, data collection on patient perspectives, health promotion and education, and offer telemedicine for online consultations and treatments [43, 63]. Use of physician-bloggers has also risen that foster sharing of health information and marketing campaigns. Cognizant with this rise in usage of social media, medical educators, physicians, and students are utilizing contents of social media regardless of its accuracy and authenticity.

### Theme II: The impact of social media on medical professionalism

Research has provided compelling evidence that social media has bipolar effect on professionalism [3] and this has led to erosion of professionalism integrity [56, 64, 65]. In a multi-site survey-based study by Garg et al., most of the identified unprofessional behaviors grouped as high-risk-to-professionalism events (HRTPE) were reported by residents [50]. The investigators have detailed that HRTPE included posting identifiable patients’ demographics, a clinical or radiological image, and inappropriate pictures of intoxicated colleagues or unprofessional remarks. The study has eluded that such events pose substantial threats to the healthcare professionals and their associated institution. Laliberté at al., have cautioned that due to blurred boundaries between professional and unprofessional territories, the Facebook friendship can potentially lead to mixing of professional and personal lives [28]. The occurrence of such phenomena is more vulnerable in hospital departments that provide intense and lengthy sessions such as rehabilitation centers.

There is an apparent dissonance between the medical students’ understanding of e-professionalism while using social media and being aware of its impact of losing professional identity [31]. Social media is considered Powerful, Public and Permanent and the impact of these three Ps potentially carries risk of disseminating misleading and inaccurate information particularly if influenced by confliction and biased interests [35]. Research has diligently proven that habitual use of social networking sites adversely affects behavioural relations [66]. In addition, there are growing concerns about negative impact of social media such as extroversion, loneliness, eccentric personality characteristics and social dissonance.

Conversely, literature has reported some benefits of use of social media by health professionals; self-directed learning by staying current, listening to patients’ opinions and needs, and patient education can potentially lead to better patient care [27, 33, 37, 41, 43, 44]. The use of social media offers valid opportunities to enhance engagement, effective feedback, professional collaboration and competence [40]. Chretien et al., have reported that, using social media, the medical students seemingly benefit from listening to patient perspectives and tend to embrace deeper cultural knowledge [26]. At the same time, using virtual patient communities on various interfaces of social media can enrich the students’ understanding of the patient perspectives [27]. Interestingly, non-hand held devices (desktop, laptop) have been shown to have a better impact on development of professional values and behaviors than hand-held devices (mobile phone, iPad, tablets) [56].

### Theme III: Blurring of professional values, behaviors, and identity in the digital era

Our review has identified a wealth of unprofessional behaviors that the researchers have welded with social media in the digital world. These include, but not limited to, indecorous description roles of pharmacists, breaches in the code of patient privacy, and offensive promotion of pharmaceutical products [20]. Profanity, sexually explicit conducts, derogatory remarks, patient demeaning, references of racism and ethnics, are some other unprofessional behaviors that have been reported in social media [51]. Physicians mostly publish pictures or other information about their patients on social media and approximately only 5 % of them obtain formal permission from their patients prior to posting [22]. Interestingly, a study has reported that 89 % physicians believed better quality of care for their patients who are connected to them through Facebook versus other patients [47]. In a survey-based investigation Marnocha et al., the authors have described that out of 293 nursing students, 77 % had encountered at least one event of unprofessional content posted by fellow students [48]. Besides, the most recurring types of unprofessional remarks were posted about patients, peers, their work environment (58 %), profanity (37 %), patient privacy (31 %), prejudicial language (29 %) and cyberbullying (11 %). This study has signaled a rising prevalence of unprofessional online behaviours by nursing students and have emphasized the crucial role of policies and formal training of digital professionalism among nursing professionals and educators.

In a report by Lefebvre et al., as many as 80 % of digitally natives were not concerned about patients’ online privacy or data protection [29]. The same report has revealed that a highest number of nurses in 36–45 years age group believed that making a patient friend or from patient’s family on social media is acceptable. Expression of humor online is considered to be more perilous than face-to-face conversations with patients. Fraping, deliberate posting of inappropriate material on Facebook, after hacking into someone else’s account is a new emerging cyber-crime [11]. This bring up another aspect of security of cyber networking that can safe-guard public’s privacy and self-esteem [32]. A study has shown extremely poor awareness about privacy regulations of social media among surgical trainees and established surgeons [35]. Furthermore, Facebook owners can access all data of their clients that they have uploaded for personal or corporate use. Lastly, a past history of posting unprofessional content on Facebook strongly predicts the occurrence of same event in future as well [52]. In other words, future professional behavior is predicted by past behaviors.

Social media notifications are a constant source for distraction and stress. Even on silent mode, haptic alerts can still arrive and cause distraction [29]. All types of notifications such as visual, auditory, or haptic lead to leads to poor work efficiency and memory. Additionally, media updates dismantle emotional states and amplifies stress levels [28]. Currently, this negative impact of social media on healthcare professionals’ health and well-being is essentially ignored.

The advantages of using social media with positive professional behaviors include sharing patient empathy, effective patient management, online publication of recommended dosage and side-effects of drugs, and rebuttal of misleading health information [20, 26]. A study has shown a gradual increase in medical students’ understanding towards considering a change in their online professional behavior [5]. This change can be attributed to increasing awareness about the deleterious effects of social media. The growing knowledge about ethical and moral use of social media can enhance its positive impact in the medical profession [50, 54].

Maintaining professional identity on social media is a daunting task as the boundary between professionalism and unprofessional conduct is delicate and invisible. Educators find it hard to define the extent to which the online identity should be allowed to reflect the concerned professional [49]. An interesting term of a “dual-citizen model” has been coined that can be applied by creating different online profiles. In a study, the participants were able to generate three leading themes that the authors argued to incorporate into existing curricula; “negotiating identities”, “maintaining distance” and “recognizing and minimizing risks” [55]. Negotiating identities as students were placed in learning climate without any role in patient care; maintaining distance by separating two crucial but unique roles; and recognizing and minimizing risks by being vigilant to new roles where professionalism might be compromised by social media. This approach can make students aware of their transitional status during their studies that will potentially mitigate risks from consequences of possible transgressions.

### Theme IV: Teaching and assessing professionalism in the digital era

Educators have advocated the incorporation of student-centered domains for social media in teaching and assessment [20]. Since the landscape of social media climate is continuously changing, there is a need for reciprocal curricular interventions to harmonize educational impact in academic institutions. The ensuing adult and active learning would aim at developing professional identities of healthcare professionals and students [5]. A great majority of medical educators and health policymakers have argued that the use of social media in medical disciplines should be taught early in medical education, and this module should include; professional standards for the use of social media, integration of social media into clinical practice, professional networking [49], and research [27]. Furthermore, essential remedial measures should be inculcated into this module that can explain concerns about social media and professionalism [29, 48]. These interventional processes would require multidisciplinary and cross-sectoral input from patients, academic and physician leaders, social media experts, and interprofessional stakeholders [34]. Finally, institutional policies about online privacy, maintaining digital professionalism, protecting medical information of patients, and the sanctions for breaching the policies should be developed and implemented. Research has shown that prior familiarity with social media policies was positively correlated with improved academic performance during online professionalism [55]. This sheds light on the educational awareness of healthcare professionals and students about online professionalism.

Social media guidance, particularly about the elements of patient confidentiality and privacy is crucial for its appropriate implementation. This may highlight the need to educate all stakeholders for essential self-disclosure for using social media [31]. Taking an oath or signing a declaration by healthcare professionals and students for adhering to the policies and regulations guidance about social media is a valid but difficult-to-implement option [11].

In summary, few institutions have incorporated a structured module of e-professionalism in their curricula despite a staggering rise in the use of social media for networking and education. Currently, academic institutional responses to cyber irregularities have typically taken the form of disciplinary actions with punitive intent. This has unintentionally created a hidden curriculum of digital unprofessionalism. This study calls for assistance and guidance in training the digitally enhanced learning in preparation for their future digitally driven clinical practice. Due to the multi-dimensional construct of professionalism, it is hard to assess all domains in the medical field. To add to its complexity, the assessment of digital professionalism is still in its infancy. This review could not identify a reliable and standard assessment tool. However, few studies have indicated initial success by comparing the posts, tweets, content, privacy settings, and personal disclosures using pre-post-intervention [34] and a longitudinal follow-up [52] with time and seniority.

### Study limitations

The term “digital professionalism” is a relatively recent term, and researchers have been writing about medical students’ and professionals’ behaviour in social media for more than 15 years. Despite these efforts, the quality of research has been found to be of low quality. Secondly, the study was not limited to one study level and has included all healthcare practitioners across the continuum. Furthermore, the studies included in this review were heterogeneous and hence we were not able to investigate the structural differences of the 44 studies due to variations in the type of information provided.

## Conclusions

Our systematic review reports an escalating rise of use of social media among health care professionals and students. This study signals unprofessional behaviors on social media among healthcare professionals. Current body of literature reports a high prevalence of breach of patient confidentiality due to an absence of existing social media policies. Nevertheless, there is a corresponding but less strong surge of awareness about the adverse effects of social media. The dignified and noble medical professionals’ identity is blurred and hazy in this digital era. Since the climate of social media is rapidly transforming, there is a need for corresponding curricular modifications that can balance legal and effective use of social media in medical education. This intervention can potentially resurrect professional virtues, behaviors and identities of healthcare professionals and students. This study highlights the need to adapt a unified policy for usage of social media among health professionals and students that can mitigate the risk of cyberbullying, patient confidentiality and professional integrity.

## Supplementary Information

**Additional file 1.** 

**Additional file 2.**

## Data Availability

All data generated or analysed during this study are included in this submitted article as Appendix I and II.

## Abbreviations

SPIDER: Sample, Phenomenon of Interest, Design, Evaluation and Research type
PRISMA: Preferred Reporting Items for Systematic Reviews and Meta-analyses
MERSQI: Medical Education Research Study Quality Instrument
HRTPE: High-risk-to-professionalism events
