# Supplementary material for: Preserving professional identities, behaviors, and values in digital professionalism using social networking sites; a systematic review
Source: BMC Med Educ. 2021 Jul 12;21:381. doi: 10.1186/s12909-021-02802-9 (PMC8273947; doi:10.1186/s12909-021-02802-9)
Supplement: Supplementary file 1 — Additional file 1. [file 12909_2021_2802_MOESM1_ESM.docx]

**Search strategy –**

**PUBMED 12^th^ May 2020 (n = 1388)**

Search: **(((((((Professionalism) OR ("professionalism"[MeSH Terms] OR "professionalism/education"[MeSH Terms] OR "professionalism/ethics"[MeSH Terms] OR "professionalism/trends"[MeSH Terms] OR "professionalism/standards"[MeSH Terms])) OR (professional attributes)) OR (professional values)) OR (professional behaviors)) OR (professional identity)) AND ((((social media) OR ("social media"[MeSH Terms] OR "social media/ethics"[MeSH Terms] OR "social media/standards"[MeSH Terms] OR "social networking"[MeSH Terms] OR "social networking/ethics"[MeSH Terms])) OR (Twitter)) OR (facebook))) AND (health professionals)** Filters: **Journal Article, from 2015 - 2020**

(((((((((((((((("professional"[All Fields] OR "professional s"[All Fields]) OR "professionalism"[MeSH Terms]) OR "professionalism"[All Fields]) OR "professionality"[All Fields]) OR "professionalization"[All Fields]) OR "professionalize"[All Fields]) OR "professionalized"[All Fields]) OR "professionalizing"[All Fields]) OR "professionally"[All Fields]) OR "professionals"[All Fields]) OR (((("professionalism"[MeSH Terms] OR "professionalism/education"[MeSH Terms]) OR "professionalism/ethics"[MeSH Terms]) OR "professionalism/trends"[MeSH Terms]) OR "professionalism/standards"[MeSH Terms])) OR ((((((((((("professional"[All Fields] OR "professional s"[All Fields]) OR "professionalism"[MeSH Terms]) OR "professionalism"[All Fields]) OR "professionality"[All Fields]) OR "professionalization"[All Fields]) OR "professionalize"[All Fields]) OR "professionalized"[All Fields]) OR "professionalizing"[All Fields]) OR "professionally"[All Fields]) OR "professionals"[All Fields]) AND (((((((("attributable"[All Fields] OR "attribute"[All Fields]) OR "attribute s"[All Fields]) OR "attributed"[All Fields]) OR "attributes"[All Fields]) OR "attributing"[All Fields]) OR "attribution"[All Fields]) OR "attributional"[All Fields]) OR "attributions"[All Fields]))) OR ((((((((((("professional"[All Fields] OR "professional s"[All Fields]) OR "professionalism"[MeSH Terms]) OR "professionalism"[All Fields]) OR "professionality"[All Fields]) OR "professionalization"[All Fields]) OR "professionalize"[All Fields]) OR "professionalized"[All Fields]) OR "professionalizing"[All Fields]) OR "professionally"[All Fields]) OR "professionals"[All Fields]) AND ("value"[All Fields] OR "values"[All Fields]))) OR ((((((((((("professional"[All Fields] OR "professional s"[All Fields]) OR "professionalism"[MeSH Terms]) OR "professionalism"[All Fields]) OR "professionality"[All Fields]) OR "professionalization"[All Fields]) OR "professionalize"[All Fields]) OR "professionalized"[All Fields]) OR "professionalizing"[All Fields]) OR "professionally"[All Fields]) OR "professionals"[All Fields]) AND ((((((((((((((((("behavior"[MeSH Terms] OR "behavior"[All Fields]) OR "behavioral"[All Fields]) OR "behavioural"[All Fields]) OR "behavior s"[All Fields]) OR "behaviorally"[All Fields]) OR "behaviour"[All Fields]) OR "behaviourally"[All Fields]) OR "behaviours"[All Fields]) OR "behaviors"[All Fields]) OR "pattern"[All Fields]) OR "pattern s"[All Fields]) OR "patternability"[All Fields]) OR "patternable"[All Fields]) OR "patterned"[All Fields]) OR "patterning"[All Fields]) OR "patternings"[All Fields]) OR "patterns"[All Fields]))) OR ((((((((((("professional"[All Fields] OR "professional s"[All Fields]) OR "professionalism"[MeSH Terms]) OR "professionalism"[All Fields]) OR "professionality"[All Fields]) OR "professionalization"[All Fields]) OR "professionalize"[All Fields]) OR "professionalized"[All Fields]) OR "professionalizing"[All Fields]) OR "professionally"[All Fields]) OR "professionals"[All Fields]) AND ("identities"[All Fields] OR "identity"[All Fields]))) AND ((((("social media"[MeSH Terms] OR ("social"[All Fields] AND "media"[All Fields])) OR "social media"[All Fields]) OR (((("social media"[MeSH Terms] OR "social media/ethics"[MeSH Terms]) OR "social media/standards"[MeSH Terms]) OR "social networking"[MeSH Terms]) OR "social networking/ethics"[MeSH Terms])) OR (("twitter"[All Fields] OR "twitter s"[All Fields]) OR "twitters"[All Fields])) OR ("facebook "[All Fields]))) AND (((("health personnel"[MeSH Terms] OR ("health"[All Fields] AND "personnel"[All Fields])) OR "health personnel"[All Fields]) OR ("health"[All Fields] AND "professionals"[All Fields])) OR "health professionals"[All Fields])

**Translations**

**Professionalism:** "professional"[All Fields] OR "professional's"[All Fields] OR "professionalism"[MeSH Terms] OR "professionalism"[All Fields] OR "professionality"[All Fields] OR "professionalization"[All Fields] OR "professionalize"[All Fields] OR "professionalized"[All Fields] OR "professionalizing"[All Fields] OR "professionally"[All Fields] OR "professionals"[All Fields]

**professional:** "professional"[All Fields] OR "professional's"[All Fields] OR "professionalism"[MeSH Terms] OR "professionalism"[All Fields] OR "professionality"[All Fields] OR "professionalization"[All Fields] OR "professionalize"[All Fields] OR "professionalized"[All Fields] OR "professionalizing"[All Fields] OR "professionally"[All Fields] OR "professionals"[All Fields]

**attributes:** "attributable"[All Fields] OR "attribute"[All Fields] OR "attribute's"[All Fields] OR "attributed"[All Fields] OR "attributes"[All Fields] OR "attributing"[All Fields] OR "attribution"[All Fields] OR "attributional"[All Fields] OR "attributions"[All Fields]

**professional:** "professional"[All Fields] OR "professional's"[All Fields] OR "professionalism"[MeSH Terms] OR "professionalism"[All Fields] OR "professionality"[All Fields] OR "professionalization"[All Fields] OR "professionalize"[All Fields] OR "professionalized"[All Fields] OR "professionalizing"[All Fields] OR "professionally"[All Fields] OR "professionals"[All Fields]

**values:** "value"[All Fields] OR "values"[All Fields]

**professional:** "professional"[All Fields] OR "professional's"[All Fields] OR "professionalism"[MeSH Terms] OR "professionalism"[All Fields] OR "professionality"[All Fields] OR "professionalization"[All Fields] OR "professionalize"[All Fields] OR "professionalized"[All Fields] OR "professionalizing"[All Fields] OR "professionally"[All Fields] OR "professionals"[All Fields]

**behaviors:** "behavior"[MeSH Terms] OR "behavior"[All Fields] OR "behavioral"[All Fields] OR "behavioural"[All Fields] OR "behavior's"[All Fields] OR "behaviorally"[All Fields] OR "behaviour"[All Fields] OR "behaviourally"[All Fields] OR "behaviours"[All Fields] OR "behaviors"[All Fields] OR "pattern"[All Fields] OR "pattern's"[All Fields] OR "patternability"[All Fields] OR "patternable"[All Fields] OR "patterned"[All Fields] OR "patterning"[All Fields] OR "patternings"[All Fields] OR "patterns"[All Fields]

**professional:** "professional"[All Fields] OR "professional's"[All Fields] OR "professionalism"[MeSH Terms] OR "professionalism"[All Fields] OR "professionality"[All Fields] OR "professionalization"[All Fields] OR "professionalize"[All Fields] OR "professionalized"[All Fields] OR "professionalizing"[All Fields] OR "professionally"[All Fields] OR "professionals"[All Fields]

**identity:** "identities"[All Fields] OR "identity"[All Fields]

**social media:** "social media"[MeSH Terms] OR ("social"[All Fields] AND "media"[All Fields]) OR "social media"[All Fields]

**Twitter:** "twitter"[All Fields] OR "twitter's"[All Fields] OR "twitters"[All Fields]

**facebook:** "facebook"[All Fields] OR "facebook's"[All Fields]

**health professionals:** "health personnel"[MeSH Terms] OR ("health"[All Fields] AND "personnel"[All Fields]) OR "health personnel"[All Fields] OR ("health"[All Fields] AND "professionals"[All Fields]) OR "health professionals"[All Fields]

**ProQuest 13^th^ May 2020 (n = 1348)**

| # | Searches | Results |
| --- | --- | --- |
| S1 | Professionalism | [**209,963**](https://proxy.rcsibahrainelibrary.com/MuseSessionID=0x106kvm1/MuseProtocol=https/MuseHost=search.proquest.com/MusePath/recentsearches.recentsearchtabview.recentsearchesgridview.scrolledrecentsearchlist.checkdbssearchlink_0:rerunsearch/A8EC001491DC4F63PQ/None?t:ac=RecentSearches) |
| S2 | Professional identity | [**851,518**](https://proxy.rcsibahrainelibrary.com/MuseSessionID=0x106kvm1/MuseProtocol=https/MuseHost=search.proquest.com/MusePath/recentsearches.recentsearchtabview.recentsearchesgridview.scrolledrecentsearchlist.checkdbssearchlink_0:rerunsearch/1A2409D8365E4B54PQ/None?t:ac=RecentSearches) |
| S3 | Professional behaviours | [**1,586,079**](https://proxy.rcsibahrainelibrary.com/MuseSessionID=0x106kvm1/MuseProtocol=https/MuseHost=search.proquest.com/MusePath/recentsearches.recentsearchtabview.recentsearchesgridview.scrolledrecentsearchlist.checkdbssearchlink_0:rerunsearch/EB5896AA3A0B4FEFPQ/None?t:ac=RecentSearches) |
| S4 | Professional values | [1,902,786](https://proxy.rcsibahrainelibrary.com/MuseSessionID=0x106kvm1/MuseProtocol=https/MuseHost=search.proquest.com/MusePath/recentsearches.recentsearchtabview.recentsearchesgridview.scrolledrecentsearchlist.checkdbssearchlink_0:rerunsearch/A664CA751DBA49D6PQ/None?t:ac=RecentSearches) |
| S5 | Professional ethics | [**722,966**](https://proxy.rcsibahrainelibrary.com/MuseSessionID=0x106kvm1/MuseProtocol=https/MuseHost=search.proquest.com/MusePath/recentsearches.recentsearchtabview.recentsearchesgridview.scrolledrecentsearchlist.checkdbssearchlink_0:rerunsearch/D0C9365C802043EDPQ/None?t:ac=RecentSearches) |
| S6 | Social media | [**1,820,584**](https://proxy.rcsibahrainelibrary.com/MuseSessionID=0x106kvm1/MuseProtocol=https/MuseHost=search.proquest.com/MusePath/recentsearches.recentsearchtabview.recentsearchesgridview.scrolledrecentsearchlist.checkdbssearchlink_0:rerunsearch/E6945F791F564793PQ/None?t:ac=RecentSearches) |
| S7 | Social networking sites | [**164,903**](https://proxy.rcsibahrainelibrary.com/MuseSessionID=0x106kvm1/MuseProtocol=https/MuseHost=search.proquest.com/MusePath/recentsearches.recentsearchtabview.recentsearchesgridview.scrolledrecentsearchlist.checkdbssearchlink_0:rerunsearch/D339923F65884BECPQ/None?t:ac=RecentSearches) |
| S8 | Twitter | [**332,511**](https://proxy.rcsibahrainelibrary.com/MuseSessionID=0x106kvm1/MuseProtocol=https/MuseHost=search.proquest.com/MusePath/recentsearches.recentsearchtabview.recentsearchesgridview.scrolledrecentsearchlist.checkdbssearchlink_0:rerunsearch/A3EB179D6614431FPQ/None?t:ac=RecentSearches) |
| S9 | Facebook | [**255,642**](https://proxy.rcsibahrainelibrary.com/MuseSessionID=0x106kvm1/MuseProtocol=https/MuseHost=search.proquest.com/MusePath/recentsearches.recentsearchtabview.recentsearchesgridview.scrolledrecentsearchlist.checkdbssearchlink_0:rerunsearch/A05D57F330F94C91PQ/None?t:ac=RecentSearches) |
| S10 | Health professionals | [2,722,521](https://proxy.rcsibahrainelibrary.com/MuseSessionID=0x106kvm1/MuseProtocol=https/MuseHost=search.proquest.com/MusePath/recentsearches.recentsearchtabview.recentsearchesgridview.scrolledrecentsearchlist.checkdbssearchlink_0:rerunsearch/70B718EFEFEA4A94PQ/None?t:ac=RecentSearches) |
| S11 | professionalism OR (professional identity) OR (professional behaviors) OR (professional values) OR (professional ethics) | [**2,394,519**](https://proxy.rcsibahrainelibrary.com/MuseSessionID=0x106kvm1/MuseProtocol=https/MuseHost=search.proquest.com/MusePath/recentsearches.recentsearchtabview.recentsearchesgridview.scrolledrecentsearchlist.checkdbssearchlink_0:rerunsearch/93587B0ADEF54009PQ/None?t:ac=RecentSearches) |
| S12 | professionalism AND (professionalism OR (professional identity) OR (professional behaviors) OR (professional values) OR (professional ethics)) | [**209,959**](https://proxy.rcsibahrainelibrary.com/MuseSessionID=0x106kvm1/MuseProtocol=https/MuseHost=search.proquest.com/MusePath/recentsearches.recentsearchtabview.recentsearchesgridview.scrolledrecentsearchlist.checkdbssearchlink_0:rerunsearch/1EA55813B3514FA4PQ/None?t:ac=RecentSearches) |
| S13 | (social media) OR (social networking sites) OR Twitter OR facebook | [**2,160,035**](https://proxy.rcsibahrainelibrary.com/MuseSessionID=0x106kvm1/MuseProtocol=https/MuseHost=search.proquest.com/MusePath/recentsearches.recentsearchtabview.recentsearchesgridview.scrolledrecentsearchlist.checkdbssearchlink_0:rerunsearch/120ACD5DD3174921PQ/None?t:ac=RecentSearches) |
| S14 | (social media) AND ((social media) OR (social networking sites) OR Twitter OR facebook) | [**2,160,035**](https://proxy.rcsibahrainelibrary.com/MuseSessionID=0x106kvm1/MuseProtocol=https/MuseHost=search.proquest.com/MusePath/recentsearches.recentsearchtabview.recentsearchesgridview.scrolledrecentsearchlist.checkdbssearchlink_0:rerunsearch/120ACD5DD3174921PQ/None?t:ac=RecentSearches) |
| S15 | (professionalism AND (professionalism OR (professional identity) OR (professional behaviors) OR (professional values) OR (professional ethics))) AND ((social media) AND ((social media) OR (social networking sites) OR Twitter OR facebook)) AND (health professionals) - | [**91,368**](https://proxy.rcsibahrainelibrary.com/MuseSessionID=0x106kvm1/MuseProtocol=https/MuseHost=search.proquest.com/MusePath/recentsearches.recentsearchtabview.recentsearchesgridview.scrolledrecentsearchlist.checkdbssearchlink_0:rerunsearch/60613F9916954142PQ/None?t:ac=RecentSearches) |
| S16 | [(professionalism AND (professionalism OR (professional identity) OR (professional behaviors) OR (professional values) OR (professional ethics))) AND ((social media) AND ((social media) OR (social networking sites) OR Twitter OR facebook)) AND (health professionals)](https://proxy.rcsibahrainelibrary.com/MuseSessionID=0x106kvm1/MuseProtocol=https/MuseHost=search.proquest.com/MusePath/recentsearches.recentsearchtabview.recentsearchesgridview.scrolledrecentsearchlist.checkdbssearchlink:rerunsearch/D81272E095174A5APQ/None?t:ac=RecentSearches) - | 4448 |

Databases:

- Ebook Central
- Health Research Premium Collection
- ProQuest Dissertations & Theses Global
- Narrowed by:
- Entered date:  2015-05-13 - 2020-05-13;
  Subject:  higher education; nursing; health education; medicine; students; medical personnel; health care; pedagogy; professionals; nurses; behavioral psychology; health sciences; hospitals; questionnaires; medical research; instructional design; medical education; attitudes; medical ethics;
  Language:  English

**ISI Web of Science (n = 50)**

| # | Searches | Results |
| --- | --- | --- |
| #1 | TOPIC:  (professionalism)  *Indexes=SCI-EXPANDED, CPCI-S Timespan=All years* | [6,813](http://apps.webofknowledge.com/summary.do?product=WOS&doc=1&qid=1&SID=8ABAyNeBOoKLovQUCWX&search_mode=GeneralSearch&update_back2search_link_param=yes) |
| #2 | TOPIC:  (professional identity)  *Indexes=SCI-EXPANDED, CPCI-S Timespan=All years* | [4,092](http://apps.webofknowledge.com/summary.do?product=WOS&doc=1&qid=2&SID=8ABAyNeBOoKLovQUCWX&search_mode=GeneralSearch&update_back2search_link_param=yes) |
| #3 | TOPIC:  (professional behaviours)  *Indexes=SCI-EXPANDED, CPCI-S Timespan=All years* | [20,587](http://apps.webofknowledge.com/summary.do?product=WOS&doc=1&qid=3&SID=8ABAyNeBOoKLovQUCWX&search_mode=GeneralSearch&update_back2search_link_param=yes) |
| #4 | TOPIC:  (professional ethics)  *Indexes=SCI-EXPANDED, CPCI-S Timespan=All years* | [7,191](http://apps.webofknowledge.com/summary.do?product=WOS&doc=1&qid=4&SID=8ABAyNeBOoKLovQUCWX&search_mode=GeneralSearch&update_back2search_link_param=yes) |
| #5 | TOPIC:  (professional values)  *Indexes=SCI-EXPANDED, CPCI-S Timespan=All years* | [20,955](http://apps.webofknowledge.com/summary.do?product=WOS&doc=1&qid=5&SID=8ABAyNeBOoKLovQUCWX&search_mode=GeneralSearch&update_back2search_link_param=yes) |
| #6 | TOPIC:  (social media)  *Indexes=SCI-EXPANDED, CPCI-S Timespan=All years* | [38,891](http://apps.webofknowledge.com/summary.do?product=WOS&doc=1&qid=6&SID=8ABAyNeBOoKLovQUCWX&search_mode=GeneralSearch&update_back2search_link_param=yes) |
| #7 | TOPIC:  (social networking sites)  *Indexes=SCI-EXPANDED, CPCI-S Timespan=All years* | [6,477](http://apps.webofknowledge.com/summary.do?product=WOS&doc=1&qid=7&SID=8ABAyNeBOoKLovQUCWX&search_mode=GeneralSearch&update_back2search_link_param=yes) |
| #8 | TOPIC:  (Twitter)  *Indexes=SCI-EXPANDED, CPCI-S Timespan=All years* | [12,217](http://apps.webofknowledge.com/summary.do?product=WOS&doc=1&qid=8&SID=8ABAyNeBOoKLovQUCWX&search_mode=GeneralSearch&update_back2search_link_param=yes) |
| #9 | TOPIC:  (Facebook)  *Indexes=SCI-EXPANDED, CPCI-S Timespan=All years* | [9,119](http://apps.webofknowledge.com/summary.do?product=WOS&doc=1&qid=9&SID=8ABAyNeBOoKLovQUCWX&search_mode=GeneralSearch&update_back2search_link_param=yes) |
| #10 | TOPIC:  (health professionals)  *Indexes=SCI-EXPANDED, CPCI-S Timespan=All years* | [102,885](http://apps.webofknowledge.com/summary.do?product=WOS&doc=1&qid=10&SID=8ABAyNeBOoKLovQUCWX&search_mode=GeneralSearch&update_back2search_link_param=yes) |
| #11 | #5 OR #4 OR #3 OR #2 OR #1  *Indexes=SCI-EXPANDED, CPCI-S Timespan=All years* | [53,046](http://apps.webofknowledge.com/summary.do?product=WOS&doc=1&qid=11&SID=8ABAyNeBOoKLovQUCWX&search_mode=CombineSearches&update_back2search_link_param=yes) |
| #12 | #11 AND #1  *Indexes=SCI-EXPANDED, CPCI-S Timespan=All years* | [6,813](http://apps.webofknowledge.com/summary.do?product=WOS&doc=1&qid=12&SID=8ABAyNeBOoKLovQUCWX&search_mode=CombineSearches&update_back2search_link_param=yes) |
| #13 | #9 OR #8 OR #7 OR #6  *Indexes=SCI-EXPANDED, CPCI-S Timespan=All years* | [52,490](http://apps.webofknowledge.com/summary.do?product=WOS&doc=1&qid=13&SID=8ABAyNeBOoKLovQUCWX&search_mode=CombineSearches&update_back2search_link_param=yes) |
| #14 | #13 AND #6  *Indexes=SCI-EXPANDED, CPCI-S Timespan=All years* | [38,891](http://apps.webofknowledge.com/summary.do?product=WOS&doc=1&qid=14&SID=8ABAyNeBOoKLovQUCWX&search_mode=CombineSearches&update_back2search_link_param=yes) |
| #15 | #14 AND #12 AND #10  *Indexes=SCI-EXPANDED, CPCI-S Timespan=All years* | 66 |
| #16 | #14 AND #12 AND #10  Refined by: PUBLICATION YEARS: ( 2020 OR 2017 OR 2019 OR 2016 OR 2018 OR 2015 )  *Indexes=SCI-EXPANDED, CPCI-S Timespan=All years* | 50 |

**Science direct/Scopus (n = 1230)**

Medical professionalism AND Social media

**EBSCO host (2015-2020) 13^th^ May 2020 (n = 39)**

Professionalism AND social media AND Health professionals
